# Flowering Phenology Adjustment and Flower Longevity in a South American Alpine Species

**DOI:** 10.3390/plants10030461

**Published:** 2021-02-28

**Authors:** Mary T. K. Arroyo, Ítalo Tamburrino, Patricio Pliscoff, Valeria Robles, Maria Colldecarrera, Pablo C. Guerrero

**Affiliations:** 1 Facultad de Ciencias, Universidad de Chile, Las Palmeras 3425, Santiago 7800003, Chile; italobioambiental@gmail.com (Í.T.); vale.robles.j@gmail.com (V.R.); 2 Instituto de Ecología y Biodiversidad (IEB), Las Palmeras 3425, Santiago 7800003, Chile; macollsu@gmail.com; 3 Departamento de Ecología, Facultad de Ciencias Biológicas, Ponticia Universidad Católica de Chile, Alameda 340, Santiago 8331150, Chile; pliscoﬀ@uc.cl; 4 Instituto de Geografía, Facultad de Historia, Geografía y Ciencia Política, Ponticia Universidad Católica de Chile, Avenida Vicuña Mackenna 4860, Santiago 7820436, Chile; 5 Center of Applied Ecology and Sustainability (CAPES), Pontificia Universidad Católica de Chile, Alameda 340, Santiago 8331150, Chile; 6 Departamento de Botánica, Facultad de Ciencias Naturales & Oceanográficas, Universidad de Concepción, Casilla 160C, Concepción 4030000, Chile; pabloguerrero@udec.cl

**Keywords:** alpine, flowering phenology, flower longevity, growing season, *Oxalis squamata*, Relative Pollination Rate index, T_BASE_, thermal growth threshold, South American Andes

## Abstract

Delayed flowering due to later snowmelt and colder temperatures at higher elevations in the alpine are expected to lead to flowering phenological adjustment to prevent decoupling of peak flowering from the warmest time of the year, thereby favoring pollination. However, even if flowering is brought forward in the season at higher elevations, an elevational temperature gap is likely to remain between the high- and low-elevation populations of a species at the time these reach peak flowering on account of the atmospheric reduction in temperature with increasing elevation. The negative effect of this temperature gap on pollination could be compensated by plastically-prolonged flower life spans at higher elevations, increasing the probability of pollination. In a tightly temperature-controlled study, the flowering phenology adjustment and flower longevity compensation hypotheses were investigated in an alpine species in the Andes of central Chile. The snow free period varied from 7 to 8.2 months over 810 m elevation. Temperatures were suitable for growth on 82–98% of the snow free days. Flowering onset was temporally displaced at the rate of 4.6 d per 100 m increase in elevation and flowering was more synchronous at higher elevations. Flowering phenology was adjusted over elevation. The latter was manifest in thermal sums tending to decrease with elevation for population flowering onset, 50% flowering, and peak flowering when the lower thermal limit for growth (T_BASE_) was held constant over elevation. For T_BASE_ graded over elevation so as to reflect the growing season temperature decline, thermal sums did not vary with elevation, opening the door to a possible elevational decline in the thermal temperature threshold for growth. Potential flower longevity was reduced by passive warming and was more prolonged in natural populations when temperatures were lower, indicating a plastic trait. Pollination rates, as evaluated with the Relative Pollination Rate index (RPR), when weighted for differences in floral abundance over the flowering season, declined with elevation as did fruit set. Contrary to expectation, the life-spans of flowers at higher elevations were not more prolonged and failed to compensate for the elevational decrease in pollination rates. Although strong evidence for phenological adjustment was forthcoming, flower longevity compensation did not occur over *Oxalis squamata’s* elevational range. Thus, flower longevity compensation is not applicable in all alpine species. Comparison with work conducted several decades ago on the same species in the same area provides valuable clues regarding the effects of climate change on flowering phenology and fitness in the central Chilean alpine where temperatures have been increasing and winter snow accumulation has been declining.

## 1. Introduction

Alpine plants face rapid elevational declines in temperature [1] and spatial variation in the depth and duration of snow cover [2,3,4,5,6,7]. These factors, together, affect the time of initiation of vegetative growth and when flowering takes place in species and their constituent populations [7,8,9,10,11,12,13,14,15].

Time of flowering, central to reproductive success, is critical for successful pollination and hence seed set [16,17]. Successful pollination will depend on the degree of coupling between flowering phenology and adequate conditions for pollinator activity. This is especially so for the many outcrossing species in the alpine [18,19,20,21,22,23,24,25,26,27,28]. Delayed flowering due to later snowmelt and colder temperatures at higher elevations could push peak flowering in the many mid-season flowering species in the alpine beyond the optimal temperature conditions for pollination. Consequently, peak flowering at higher elevations is expected to be subject to forward phenological adjustment in order to avoid decoupling from the warmest time of the year. At the same time, greater flowering synchrony could be expected to assure larger floral displays. 

Even if phenological adjustment allows flowering at higher elevations to occur in the warmest time of the season in mid-season flowering species, temperatures at peak flowering are still likely to remain cooler on higher elevation sites because of the adiabatic temperature lapse rate. In this regard, flower longevity, a temperature-driven, plastic reproductive trait [23,29,30,31,32,33], is likely to come into play. Flower longevity has been shown to increase with elevation and on colder alpine microsites [9,13,34,35,36,37]. It decreases under experimental warming and in alpine plants transplanted to lower elevations [30,33,38,39]. It also responds to pollination in many species [32,40]. Many alpine species have fairly long-lived flowers [39,41] and some alpine species with intrinsically long life-spans show relatively little pollen limitation [42,43]. Moreover, plastically-elongated flower life-spans have been shown to ameliorate slower pollination rates on colder alpine microsites [30]. Prolonged flower longevity allows flowers to “sit and wait” for more time to be pollinated. Consequently, the probability with which a flower will be pollinated increases [30,31,34,44,45,46,47]. Flower longevity thus is a critical trait in environments such as the alpine, where temperature varies widely at both spatial and temporal scales and where pollinators are highly sensitive to temperature conditions [48,49]. 

The degree to which phenological adjustment and flower longevity compensation occur in different alpine systems across the globe is unknown. One comprehensive study revealed that snowbed species tend to exhibit phenological adjustment, while fell field species do not [15]. Although longer-lived flowers have often been described for higher elevations, studies are usually based on short periods of the flowering season and hence might not always be representative of the true elevation tendencies. Here, flowering phenology and flower longevity are investigated over the elevational range of *Oxalis squamata*, a self-incompatible pollinator-dependent alpine species, in the high Andes of central Chile. Although this species is not a true snowbed species, strong support for the flowering phenology adjustment hypothesis was found. Flower longevity increased with lower temperatures. However, the flower longevity compensation hypothesis was not supported. 

## 2. Materials and Methods

### 2.1. Focal Species and Study Area

**Figure 1:**
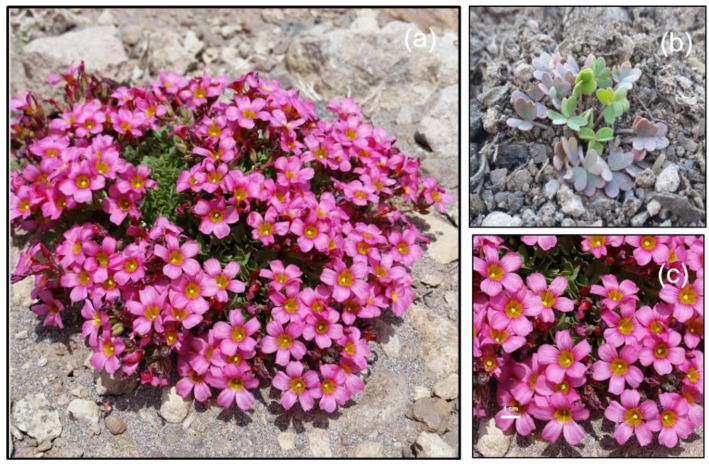
(**a**) A typical loose cushion of *Oxalis squamata* on Site III. Captured on 12 Jan 2016 by M.T.K.A. (**b**) Plant greening up on Site I. Captured on 20 August 2019 by M.T.K.A. (**c**) Details of flowers.

*O. squamata* (Oxalidaceae) is a tristylous, strongly self-incompatible, short-lived perennial herb [50,51] (Figure 1a). It is found on well-drained sites with moderate snow cover. The plants green up rapidly after snowmelt (Figure 1b). Preformed flower buds are absent. On very cold days, the flowers fail to open, but may reopen on subsequent days when temperatures rise again. Flowers are pollinated mostly by tiny, fast-moving Andrenid bees which are very difficult to observe, and occasionally by *Megachile* species [50]. The small Andrenid bees are ecothermic. They access plants from nearby rocks where they bask to gain heat. They are active only on warm days. In constantly returning to rocks for heating, their flight ranges are very limited. 

**Figure A1:**
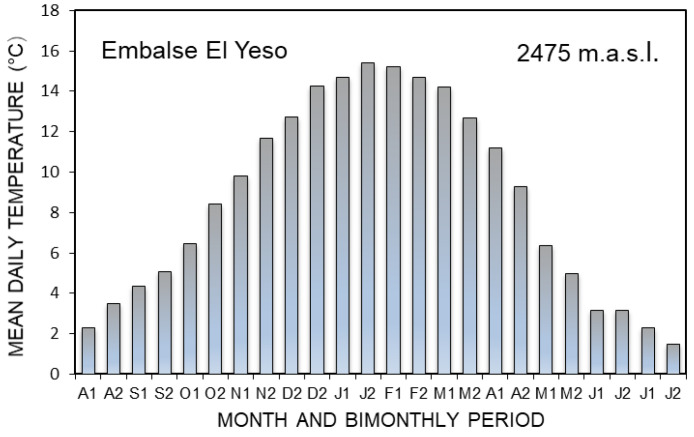
Average bimonthly air temperatures recorded by the Embalse El Yeso weather station, Metropolitan Region, Chile (33°40’36” S, 70°05’19”S W, 2475 m.a.s.l.) located in the subalpine zone, ca. 36 km to the south of the study site. Based on data available as of 1978. Source of data: CR2 Climate Explorer available at http://explorador.cr2.cl/, accessed 30 September 2020. The warmest period of the summer in the region is the second half of January. The letters on the x-axis refer to months starting in August (early spring) and ending in July (late winter).

Work was carried out in the Farellones-Valle Nevado area. Five vegetationally homogeneous sites covering a large part of *O. squamata’s* elevational range in the study area, including the highest known population, were selected (see Table 1 details). The two subalpine sites and the ecotonal site are fairly flat. The two high alpine sites are gently sloping. One faces NW (IV) and the other E (V). Mean annual temperature for the closest weather station with a fairly long record (1977 onwards, 36 km to the south) (Embalse del Yeso—33°40’36” S, 70°05’19”S W, 2475 m.a.s.l.) is 8.7 °C (CR2 Climate Explorer available at http://explorador.cr2.cl/, accessed on 30 September 2020). The second half of January is the warmest period of the summer (Figure A1). Winter snow begins to accumulate from April to June and can remain on the ground until early November, depending on elevation. The spring and summer months are mostly sunny and dry. Short-duration summer snowstorms and convective rain are occasionally received [52]. Whole season community-level flower visitation rates and individual species flower visitation rates for other species decline with elevation in the same general area [48,49].

### 2.2. Flowering Phenology 

About 30–50% of the area covered by each of the five populations was cordoned off in sampling areas. From 5 November 2016 to 28 February 2017, all plants that came into flower were marked and the number of open flowers per plant counted on multiple occasions across the flowering season mostly at 3–5 day intervals (see Table 1 for sample sizes). Only a few small plants did not come into flower. On 19% of occasions, the intervals between observation dates were longer or shorter on account of weather conditions and logistical considerations. Overall, phenological observations (eliminating a heavy snowfall day) were made on 19 to 30 separate occasions per site, giving a total of 133 site visits. Browsing animals broke through temporary fences, disturbing some plants on Sites III and IV. However, because the damage occurred fairly late in the season and all but one plant continued to flower, the damage is unlikely to have had a serious effect on the flowering metrics considered. 

The length of the growing season and flowering phenology were assessed using heat sums (accumulated growing degree days—GDD). The date of definitive snowmelt on a site was the biofix date. MODIS Terra Snow Cover data were downloaded (https://search.earthdata.nasa.gov, accessed on 6 December 2018 onwards). MODIS Terra satellite maps show % snow cover on a daily basis at the 500 m × 500 m resolution. Snowmelt dates were based on continuous 0% snow cover, allowing a 10% margin. MODIS data were also used to capture the date of the first permanent winter snow. Temperature data for the GDD calculations were obtained from the more recently established and closer Valle Olivares weather station (33°11’15” S, 70°06’44” W, 2787 m.a.s.l.) (http://explorador.cr2.cl/, accessed 14 August 2019). This station is found at a similar elevation to our middle site (III). To obtain daily temperatures for each site, the temperature difference between contig-uous pairs of sites was estimated, based on air temperatures recorded on each site at 20 min intervals at 1.5 m.a.g.l for some or all days in Nov through Mar (Sites 1-IV) and Dec through Mar (Site V) of the study year. These temperatures were recorded with data loggers (HOBO U23 Pro v2; Onset Computer Corp., Cape Cod, MA, USA). The resultant average monthly differences were added or subtracted to the weather station data to obtain daily average temperatures for each site. Prior to subtraction, a minor adjustment of −0.04°C was made to the weather data to bring it into line with the logger temperatures for Site III over the Nov-Mar period. 

Following other alpine studies, T_BASE_ fixed at 5 °C and 1 °C [12,53,54,55,56] were used. Additionally, a graded series of T_BASE_ temperatures reflecting the average daily air temperature differences between the sites over the full snow-free period of the study year was developed. Accordingly, T_BASE_ was set to 4.20 °C (Site II), 2.83 °C (Site III), 1.31 °C (Site IV), and 1.07 °C (Site V), while maintaining it at 5 °C for the lowest site (I). GDD was calculated as the sum of [(T_max_ + T_min_)/2-T_BASE_] where T_max_ and T_min_ are daily maximum and minimum temperatures (°C), respectively. When (T_max_ + T_min_)/2 was equal or fell below T_BASE_ GDD, it was set to 0.

### 2.3. Flower Longevity and Pollination Rates

To determine whether flower longevity responds plastically to temperature, flowers on 16 plants were passively warmed on two sites (I, IV) with Open Top Chambers (OTCs). Sixteen additional plants outside the OTCs served as controls. All plants were pollinator-excluded. The marked buds (3 per plant) were followed daily until flower closure. Temperature was recorded inside and outside of a number of the OTCs with data loggers placed at 15 cm.a.g.l. Additionally, a temperature-controlled survey of flower longevity in natural populations was undertaken. A targeted twelve plants per site were haphazardly chosen. Six plants were randomly assigned to a pollination-exclusion treatment designed to quantify potential flower longevity (PFL), the maximum potential life-span of a flower. The remaining six plants were left exposed to pollinators (open-pollination treatment) to determine AFL, the time a flower remains open following open-pollination. On each plant, five mature flower buds were marked. When flower buds became scarce toward the end of the flowering season, the number of buds marked was reduced to three and some plants had to be reused. Flower diameter was measured on the day of anthesis. The marked flowers were monitored daily. The temperature was recorded at 20 min intervals at 15 cm.a.g.l. with data loggers. Because flower longevity can be affected by the amount of water in the soil (38), soil moisture was measured on most of the days the flowers were open (FIELDSCOUT TDR300, Spectrum Technologies Inc., Aurora, IL, USA). Overall, 17 (I, II), 16 (III, IV), and 9 (V) flower batches were assessed for flower longevity, giving a total of 75 batches across all sites. Due to heavy snow, three batches had to be eliminated (one each on Site 1, II and V) plus some damaged and lost flowers. Flower longevity was successfully measured on a total of 1933 pollinator-excluded flowers (I = 427, II = 440, III = 429, IV= 437, V = 200) and 1841 pollinator- exposed flowers (I = 420, II = 430, III = 369, IV = 411, V = 211). 

**Figure A2:**
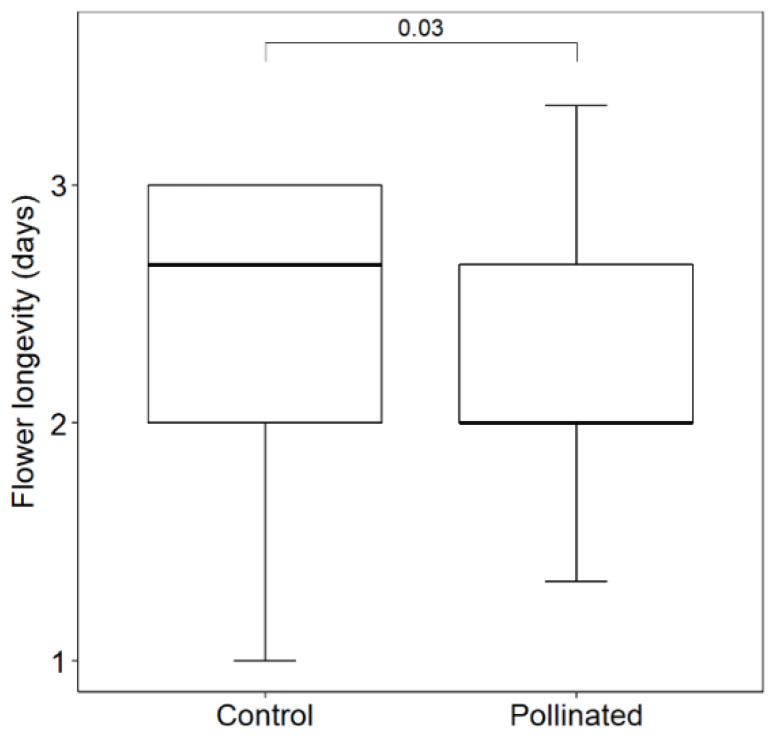
Effect of experimental hand-pollination on flower longevity in *O. squamata.* The number above the bracket gives the *p*-value according to the Mann–Whitney U-test. The experiment was carried out on Site V. Both control and pollinated flowers were pollinator-excluded.

Pollinator availability among sites was compared with the Relative Pollination Rate index (RPR). This index, given by: RPR = 1-ALF/PLF, is a proxy for the relative rates of flower visitation on a site. When pollination is slow (equivalent to low flower visitation rates), the average flower on a site will remain open for a large part of its potential life-span [30,49]. Accordingly, when ALF → PFL, RPR → 0. Values of RPR → 1 indicate AFL is relatively small in relation to PFL. In this case, the open-pollinated flowers would have received pollen comparatively early in their life-spans (equivalent to high flower visitation rates) and thus closed earlier on average with respect to their maximum potential life-spans. The RPR index relies on a demonstration that pollination provokes flower senescence. A field experiment confirmed this to be the case in *O. squamata* (see Appendix B, Figure A2).

### 2.4. Fruit and Seed set

Around 3–4 weeks after each batch of flowers was marked, fruits were collected and the seed counted in the laboratory. Pollinator-exposed flowers were used to quantify seed set via open pollination, while pollinator-excluded flowers served to check for autonomous self-pollination. As seed per fruit can be affected by ovule number in a flower, ovule number was determined. Ovule counts were made directly at the fruiting stage where possible or from pickled flowers collected on the same plants. Retrieved sample sizes excluding losses fluctuated between 180 and 398 (open-pollinated flowers) and 186 and 416 (pollinator-excluded flowers). Fruit set was the proportion of retrieved flowers per flower batch that set fruit, while seed set was the number of seed per fruit formed. 

### 2.5. Data Treatment and Statistical Analysis

#### 2.5.1. Flowering Phenology

On Sites I and V, several plants had a few flowers at the time of arrival in the field. On Site IV, where the plants were very large, some plants continued to flower at the end of February when the field work terminated. In the remaining sites, none or very few plants had a few flowers at the beginning and end of the field observations. To obtain accurate first and last flowering, second-order polynomials were fitted to the field observations, and the equations resolved to zero flowerings at the extremes (Table A1). To obtain the additional days of flowering for a particular plant that had started flowering, it was assumed that plants with large numbers of flowers had been or would have continued to be in flower for a longer period of time than plants with small numbers of flowers. Results were adopted at three-day intervals. Heavy snowfall prevented counting the flowers on one appointed date. Additionally, some of the smaller plants showed erratic flower openings. In these cases, a plant was considered to be in the flowering phenophase when flowering bracketed the problem dates. The Augspurger’s flowering synchrony index [57] was calculated to flowering overlap. 

The snow-free period and the biological relevant growing season (GS) were calculated based on GDD. To compare flowering phenology among sites, GDD was calculated over the days between the site-specific snowmelt date and the date of the event for the six phenological metrics listed below.

1. First individual that flowered (FF)
2. Half of the plants in flower (FL_50%_)
3. Peak flowering—maximum number of plants in flower (FLP)
4. Peak floral abundance—maximum number of open flowers (FLSP)
5. First flower that opened on each individual of the population (FF_POP)_
6. Peak flowering for individuals in the population based on the day of the maximum number of open flowers per plant (FLP_POP_)

GDD per site was regressed on elevation for each T_BASE_ scenario. Median values of GDD were used to represent FF_POP_ and FLP_POP_. Additionally, the effect of site was analyzed for FF_POP_ and FLP_POP_ with the Kruskall–Wallis test.

#### 2.5.2. Flower Longevity and Pollination Rates

The effect of experimental warming on flower longevity was assessed with two-way ANCOVA with flower diameter as a covariate. Although flower longevity was not normally distributed, colinearity between the covariate and the outcome variable, homogeneity of the regression slopes and normality, and homoscedasticity of the model residuals was ascertained. To avoid intraplant microclimatic positional effects on flower longevity that are typical in *Oxalis* species, the means for the flowers on each plant were used [30]. 

Factors potentially affecting flower longevity in natural populations were analyzed with mixed model ANCOVA. Batch within site was a random block effect, pollination treatment was a fixed effect, and temperature, soil moisture, and flower diameter were continuous covariates. The unit of analysis was mean flower longevity per plant. Temperature and soil moisture were the means for the days over which each individual flower on a plant remained open. When soil moisture data were missing, the closest available dates were used. Flower longevity and temperature were also compared via linear regression. Differences in PFL between sites and differences between PFL and AFL within sites were analyzed with Dunn’s test for pairwise multiple comparisons on ranked data after performing Kruskal–Wallis tests. RPR was analyzed with One-Way ANOVA.

#### 2.5.3. Fruit and Seed Set

Fruit set and ovule number per flower were analyzed with 1-Way ANOVA. Seed per fruit was analyzed with the Kruskall–Wallis test. To obtain seasonally-representative measures of RPR and fruit set, the raw data for each flower batch was weighted by floral abundance. Floral abundance was obtained from the closest phenological observation dates (usually two, sometimes one, depending on how long an individual batch of flowers remained open). Values for each batch were multiplied by floral abundance. The average across all batches was then calculated.

## 3. Results

### 3.1. Flowering Phenology

**Figure 2:**
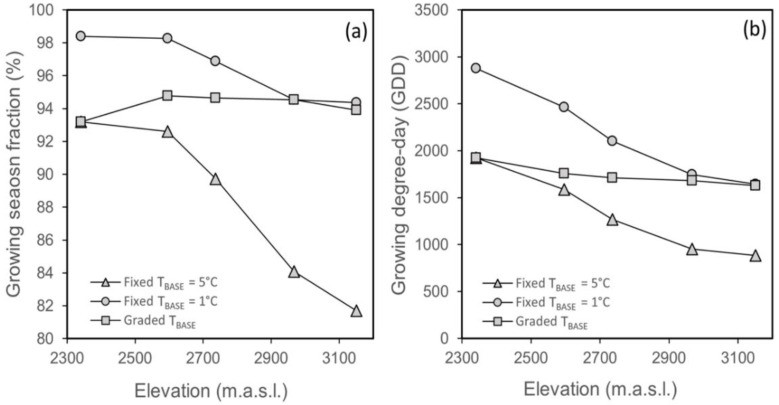
(**a**) Trends in the fraction of days corresponding to the biological growing season (GS) over 810 m of the elevational range of *O. squamata* in the central Chilean Andes according to three T_BASE_ scenarios. (**b**) Trends in accumulated growing degree days (GDD_GS_) for the same three scenarios.

The snow-free period over 810 m of *O. squamata’s* (Table 1) elevational range lasted between 8.2 and 7.0 mths depending on elevation. The elevational temperature difference over the snow-free period was 3.9 °C. Snow lifted in early spring on the lowest site (Table 1). It remained on the ground for 37 more days on the highest site (Table 1). This difference translates into an increase of 4.6 days 100 m^−1^ in permanent snow cover. The biologically-relevant growing season (hereafter, GS) was calculated for three T_BASE_ scenarios: T_BASE_ = 5 °C fixed across elevation; T_BASE_ = 1 °C fixed across elevation; T_BASE_ graded from 5 °C on Site I to 1.07 °C on Site V). Under T_BASE_ fixed at 5 °C, not surprisingly, GS was shortest but still occupied a very high fraction of the snow-free days in the upper part of the range (Figure 2a). As expected, the decline in snow-free days, together with the difference in temperature at the elevational extremes, led to declines in GDD_GS_ under both T_BASE_ scenarios (Figure 2b). The decline was steeper for Tbase = 5 °C.

Under the hypothetical scenario of a progressive elevational reduction in T_BASE_, GS and GDD_GS_ showed less elevational variation (Figure 2a,b). The fraction of snow-free days across sites for which growth would have been possible was consistently over 90%. Overall, these results confirm the snow-free period and the biological GS on the Andean location is long. They also show that sizable elevational differences in the opportunity for growth will exist when a plant species’ lower temperature limit for growth remains invariant with elevation, potentially impacting flowering times. 

**Figure 3:**
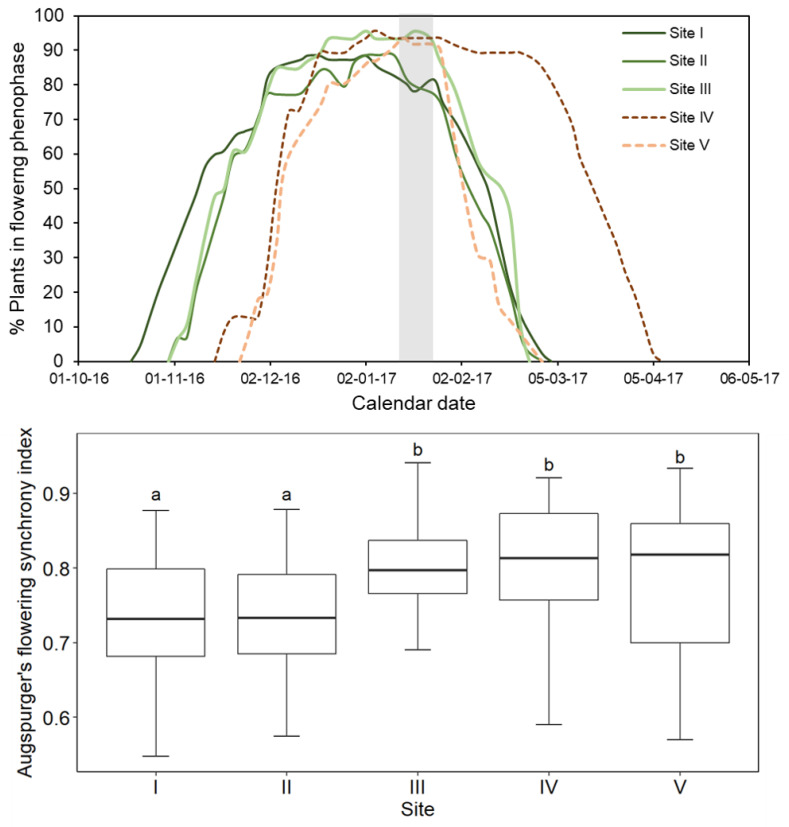
(**a**). Smoothed curves for the percentage of plants in the flowering phenophase over the day intervals (see Materials and Methods section for more details). The grey shading indicates the historically regionally-warmest half month of the summer season. See Figure A1 for more details. Flowering (FF) commenced on 21 Oct on Site I, 2 Nov on Sites II and III, 17 Nov on Site IV, and 25 Nov on Site V. Peak flowering (FLP) occurred on 14 Dec on Site I, 2 Jan on Sites II and III, 5 Jan on Site IV, and 14 Jan on Site V. (**b**). Degree of overlap (flowering synchrony) in the flowering periods of individual plants on each site according to the Augspurger flowering synchrony index. Different letters indicate significant differences according to the Dunn test at *p* < 0.05.

Flowering onset (FF) occurred earlier on the two subalpine (I, II) and ecotonal sites (III) than on the two high alpine sites (IV, V) (Figure 3a). Peak flowering (FLP) likewise occurred earlier on the two subalpine and the ecotonal site. Considering the two altitudinal extremes, the overall elevational delay for FF was 4.3 d 100 m^−1^ elevation and 3.8 d 100 m^−1^ for FLP. These differences are somewhat smaller than the 4.6 d 100 m^−1^ elevational lag in snowmelt. Site had a significant effect on flowering overlap among individuals (Kruskal–Wallis test, X^2^ = 40.312, df = 4, *p* < 0.00001). As predicted, there was a clear tendency for greater flowering overlap on the higher-elevation sites (Figure 3b). This translated to higher proportions of the plants on a site being in flower on the peak flowering dates in the higher alpine sites (Figure 3a). Based on long-term records, the warmest period of the summer in this area of the Andes occurs in the second half of January (Figure A2). Thus, *O. squamata* would be classified as an early mid-season flowering species. The historical regional temperatures corresponding to the dates of peak flowering on the uppermost and lowermost sites are 14.1 °C (Site I) and 12.1 °C (Site V) (interpolated from the Embalse del Yeso weather station and a summer lapse rate of 4.4 °C over the months of *O. squamata*´s flowering period). Thus, the elevational difference in temperature at the time of peak flowering is 2.4 °C lower than the overall lapse rate. This comparison provides a first indication that flowering phenological adjustment has occurred in *O. squamata.*

**Figure 4:**
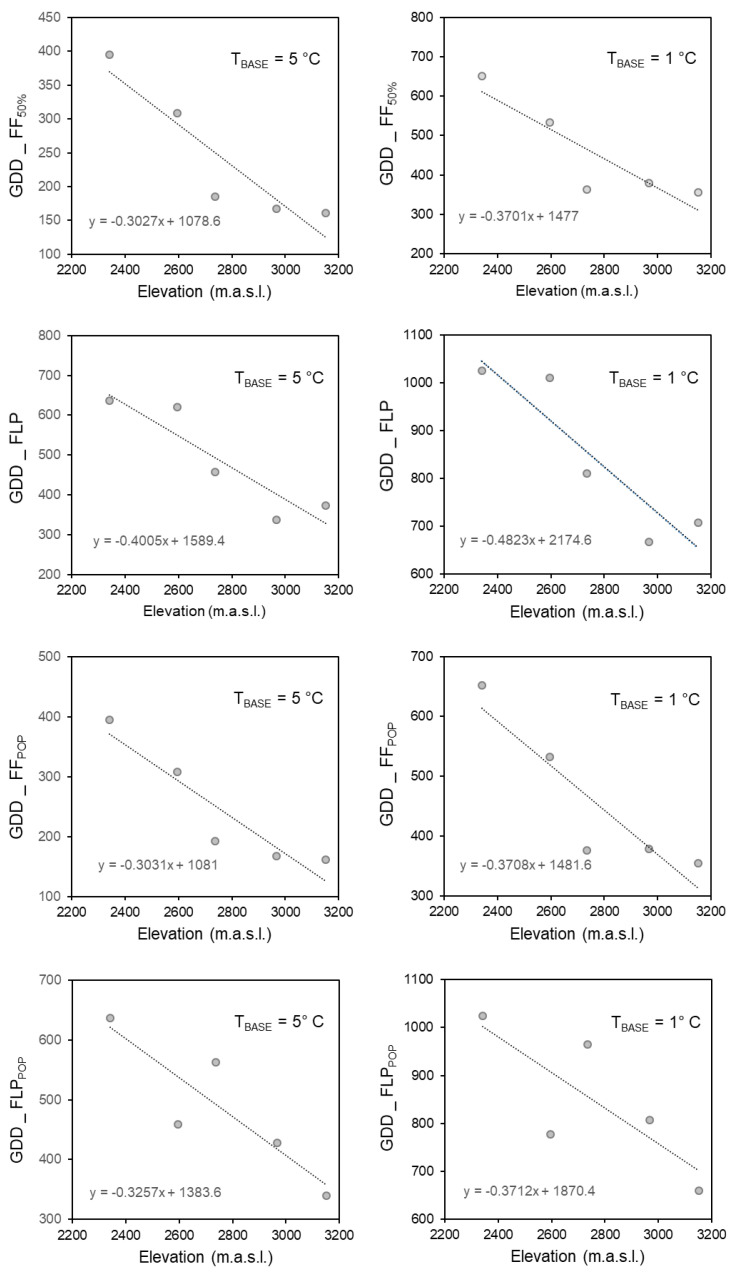
Regressions of GDD_FLP, GDD_FF_50%_, GDD_FF_POP_, and GDD_FLP_POP_ for fixed T_BASE_ = 5 °C and fixed T_BASE_ = 1 °C on elevation. See Table 2 for significance of regressions.

Mean temperature over the period from snowmelt to flowering onset (FF) declined from 8.9 °C on Site I to 5.7 °C on Site V. The corresponding temperatures at peak flowering (FPL) were 10.6 °C and 7.8 °C. A strong effect of site was found for GDD_POP__FF (T_BASE_ = 5 °C, Χ^2^ = 140.58, *p* < 0.00001; T_BASE_ = 1 °C, Χ^2^ = 103.88, *p* < 0.00001) and GDD_POP__FLP (T_BASE_ = 5 °C, Χ^2^ = 166.41, *p* < 0.0001; T_BASE_ = 1 °C, Χ^2^ = 138.8, *p* < 0.0001) (Kruskall–Wallis Test). Table 2 shows the results of the regressions of GDD on elevation for the different flowering metrics according to thermal growth threshold scenario. For T_BASE_ fixed at 5 °C and 1 °C, elevation was a significant predictor of GDD_POP__FF_,_ GDD_FF_50%_ and GDD_FLP, with GDD in all cases decreasing strongly with elevation (Figure 4). Elevation was also a significant predictor of GDD_POP__FLP for T_BASE_ = 5 °C (Figure 4). In the case of T_BASE_ = 1 °C, the trend for the last metric was in the same direction but it was not significant (Table 4). There was also a non-significant tendency for GDD_FF to decline with increasing elevation (Table 2). GDD_FLSP failed to show a clear elevational trend. Although animal damage occurred after FLP, the peak flowering dates for eight individual plants on Site IV based on flower number could have been affected and thus influenced GDD_FLP_POP_. To evaluate this possibility, the peak flowering dates were pushed back further in the season. This had no effect on the median GDD_POP__FLP values that were used in the respective regression. Overall, FLSP would also unlikely to have been affected.

For the graded series of T_BASE_ temperatures (Table 2), elevation was not a significant predictor of GDD for any phenological metric (Table 2) even though there was a significant effect of site on FF_POP_ (Kruskall–Wallis Test: Χ^2^ = 22.22, *p* < 0.0005) and FLP_POP_ (Kru–kall-Wallis Test: Χ^2^ = 70.07, *p* = 0.0001). Overall, all T_BASE_ scenarios allowed peak flowering at the higher elevations to be situated precisely around the time of summer when temperatures are highest (Figure 2, Figure A2). These results are highly consistent with the flowering phenology adjustment hypothesis. 

Finally, while there is a suggestion of an elevational decline in the number of days required to reach some of the flowering phenology phases (Table 1), the trends were not significant (regressions not shown). Thus, in the case of *O. squamata,* flowering phenological adjustment is manifest principally at a physiological level.

### 3.2. Flower Longevity and Pollination Rates

**Figure 5:**
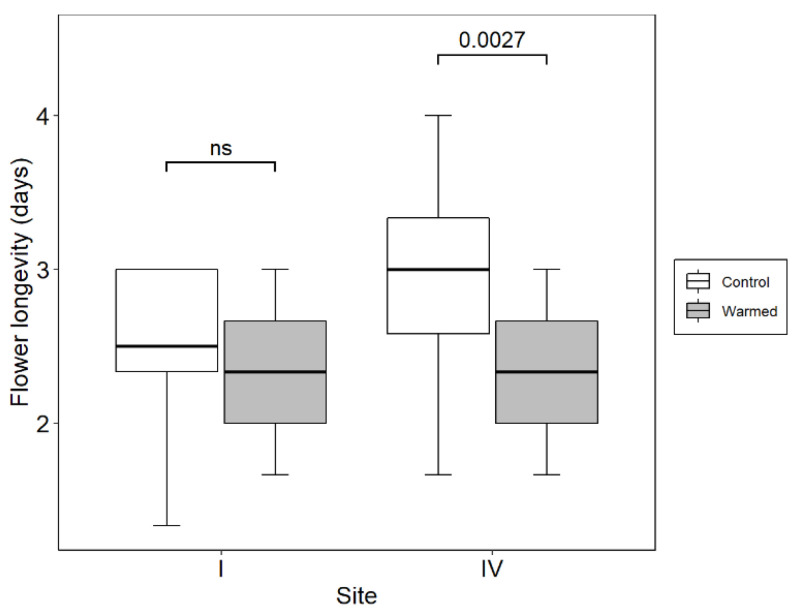
Effect of experimental warming on flower longevity in *O. squamata* on a subalpine (Site I) and a high alpine site (Site IV). The two sites are separated by 635 m elevation. The numbers above the brackets give the *p*-value according to Mann–Whitney U-test. n.s = not significant at *p* = 0.05. The open top chambers (OTCs) used to warm flowers of *O. squamata* increased temperature around the flowers by 3.9 °C (Site I) and 3.6 °C (Site IV) over the controls.

Warming had a significant effect on the potential flower life-span (Table 3). This is reflected in the treatment flowers lasting significantly less time than the control flowers on Site IV (Figure 5). However, the difference was not significant on the naturally already much warmer Site I. The marginally significant interaction between site and treatment provides evidence that the different baseline temperature conditions on the two sites were relevant in this experiment (Table 3). Flower size had no effect on flower longevity.

**Figure 6:**
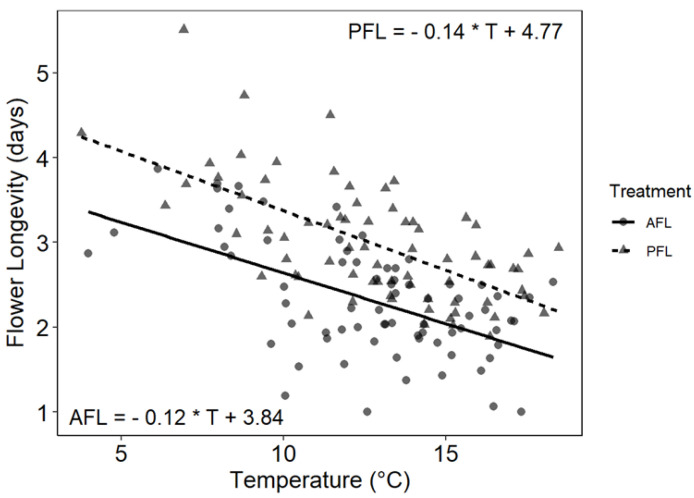
Linear regressions of flower longevity on temperature. Temperatures are for the same days the flowers in a batch remained opened. Individual points correspond to the mean flower longevity of each flower batch. Temperature was a significant predictor of both PFL and AFL (PFL: F_1,71_ = 32.211, *p* = 2.900 E-07; AFL: F_1,71_ = 46.272, *p* = 2.857 E-09).

Individual pollinator-excluded flowers (potential flower longevity, PFL) lasted 1–7 d, while open-pollinated flowers (actual flower longevity, AFL) remained open between 1–6 d. These wide ranges indicate many variations in flower longevity. Temperature and pollination treatment both had a significant effect on flower longevity (Table 4). However, again there was no effect on flower diameter. Soil moisture was not important. The relationship between flower longevity and temperature is shown graphically in Figure 6, which distinguishes between PFL and AFL. Colder temperatures were clearly associated with more prolonged flower longevity. The intercepts of the two regression lines were significantly different (Chow’s test; F_2,140_ = 27.55, *p* = 8,13E-11), indicating that AFL tends to be shorter than PFL, indicating that some pollination had taken place. Although the slope of the curve for PFL was somewhat steeper than for AFL, the two slopes were not significantly different (t_140_ = 0.180, *p* = 0.8572). The last result is not surprising given a large amount of scatter around both regression lines. The results indicate that flower longevity is more prolonged under naturally colder temperatures and thus, theoretically, could play a functional role in compensating for expected lower visitation rates at higher elevations given the remaining temperature gap at peak flowering on the extremes of *O. squamata’s* over elevational distribution. 

**Figure 7:**
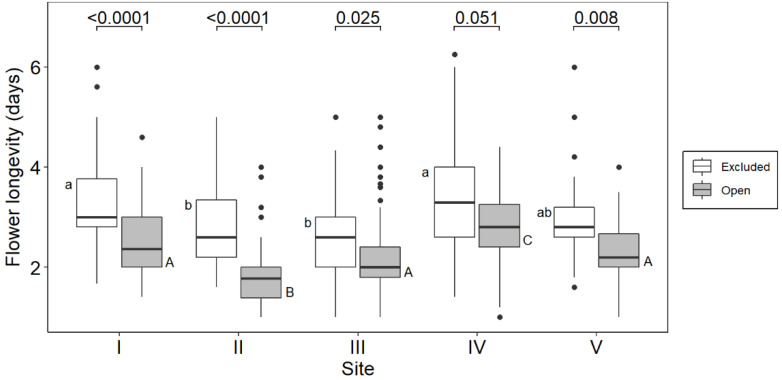
Flower longevity in pollinator-excluded (PFL) and pollinator-exposed (AFL) flowers of *O. squamata* on the five sites ordered from lower to higher elevation. See Table 1 for elevations of sites. Lower case letters alongside the PFL and upper-case letters alongside AFL boxes show significant differences between sites according to the Dunn test following analysis with the Kruskall–Wallis test (PFL: X^2^ = 52.375, *p* = 1.152e-10; AFL: X^2^ = 100.81, *p* < 2.2e-16). The numbers above the brackets are the P values for differences between AFL and PFL on each site according to the Dunn test.

PFL was expected to increase with elevation given the colder temperatures at higher elevations over *O. squamata’s* flowering period. Contrary to expectation, PFL showed no clear elevational increase (Figure 7). This continued to be true when PFL was weighted for floral abundance in order to provide a seasonal estimate for PFL (Sites I: 3.1 d, II: 2.7 d, III: 2.4 d, Site IV: 3.0 d, Site V: 2.8 d). However, AFL was always shorter than PFL, indicating that some pollination had occurred on all sites (Figure 7).

**Figure 8:**
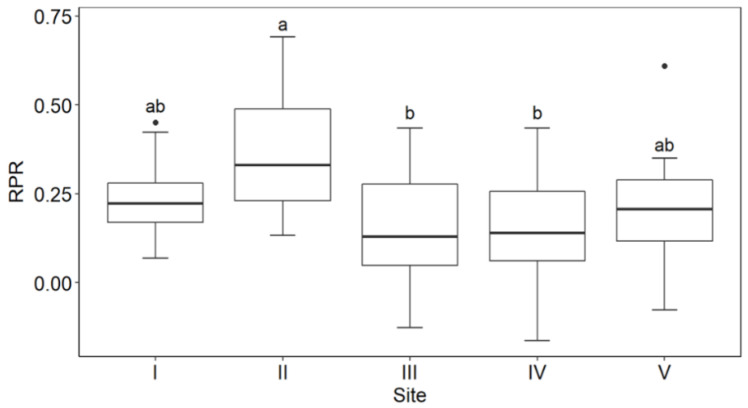
Comparison of the RPR index for *O. squamata* on the five alpine sites. Sites are ordered from the lowest to the highest-elevation site. See Table 1 for elevations of sites. Higher values of this index indicate faster pollination than lower values. Different lower-case letters indicate significant differences between sites based on the Tukey test following One-Way Anova (F_1,4_ = 3.989, *p* = 0.00581).

Although pollination had occurred on all sites, the rate of pollination as assessed by the RPR index showed no clear elevational trend (Figure 8). However, when flower longevity for each flower batch was weighted by floral abundance to obtain seasonal estimates for the sites and the index recalculated, the picture changed. The two high alpine sites now showed the lowest RPR values (Site IV: 0.114, Site V: 0.107), while the two subalpine sites had the largest values (Site I: 0.210, Site II: 0.369). The ecotonal Site III fell in between (0.115). Thus, there is strong evidence for progressively poorer pollination with increasing elevation in *O. squamata*. 

### 3.3. Fruit and Seed Set 

**Figure 9:**
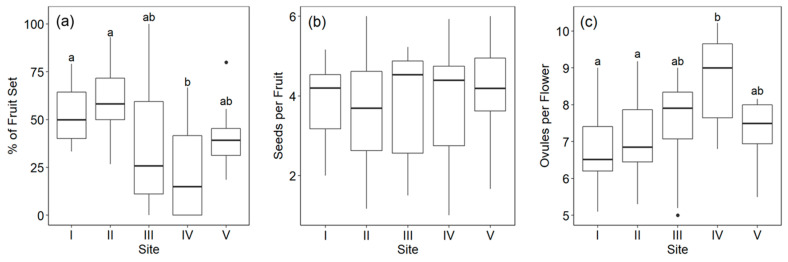
(**a**) Percent fruit set, (**b**) seed number per fruit, and (**c**) number of ovules per flower in open-pollinated plants of *O. squamata* on the five sites ordered from lower to higher elevation. See Table 1 for elevations of sites. Different letters indicate significant differences according to the Tukey a posterior test for the variables where a significant effect of site was found. Site had no significant effect on seed per fruit.

Flower longevity compensation was expected to level out fruit set across elevation. Figure 9 shows the results for fruit and seed set according to the site. Fruit set per flower batch for the open-pollinated flowers varied from 0–100%. A significant site effect was found (1-Way Anova; *p* = 0.00024) for fruit set but not for seed per fruit (Kruskall–Wallis Test, Χ^2^ = 2.282, *p* = 0.68) (Figure 9a,b). Although there was a tendency for lower fruit set in the three uppermost populations, the high variability in fruit set among the flower batches and the many lost or damaged flowers impeded good resolution except in the case of Site IV, which had a significantly lower fruit set than all other populations (Figure 9a). When fruit set was weighted by floral abundance to obtain a more realistic seasonal estimate of fitness for the sites, fruit set was highest on the two lowest sites (Site I: 51.5%, Site II: 62.8%, Site III: 28.3%, Site IV: 23.2%, Site V: 31.5%). 

Site also had a significant effect on ovule number (1-Way Anova, *p* = 0.0017). The three highest sites tended to have more ovules per flower (Figure 9c). Thus, although seed set per flower did not decline with elevation (Figure 9b), reflecting the lower pollination rates, relatively fewer ovules in a flower would have been pollinated on the higher sites. 

Only 1.1% of the total retrieved pollinator-excluded flowers formed fruits (0–1.9% among sites) compared with 44.1% of the pollinated-exposed flowers. The difference was highly significant (X^2^ = 942.69, *p* < 0.00001). Thus, the above results are not affected by autonomous self-pollination. Overall, the open-pollination fruit set is in accordance with a lack of flower longevity compensation in the focal species.

## 4. Discussion 

In this study, two hypotheses relevant to the success of plant pollination and fitness in the harsh alpine environment were tested. The phenological adjustment hypothesis, notwithstanding evident phylogenetic constraints among different taxa, posits unreliable pollination at higher colder elevations in the alpine will favor phenological adjustment to situate flowering in the warmest possible time of the year. The flower longevity compensation hypothesis sees plastically-prolonged flower longevity ameliorating lower pollination rates expected on account of a remaining temperature gap at the time of peak flowering at higher compared to lower elevations. Flowering phenology and flower longevity have been studied separately on many occasions. However, integrative studies such as reported here, are lacking. 

The snow-free period of >8 mth on the lowest site would appear to be one of the longest reported for alpine areas in warm and dry temperate areas [1,58,59,60]. The treeline in the study area, formed by an angiosperm species, is relatively low compared to typical northern hemisphere gymnosperm treelines. Additionally, the central Chilean treeline is elevationally depressed on account of aridity [61]. However, even if the lower limit of the alpine zone were pushed up to around 2700–2800 m.a.s.l. where the global bioclimatic treeline [62] is likely to lie, the snow-free period, in general, in the study area would remain exceptionally long, as has been reported in earlier studies [9]. 

Strong evidence for flowering phenological adjustment was found over 810 m of *O. squamata’s* elevational range. The first indication of such adjustment came from the smaller lag times between flowering onset and peak flowering, respectively, between the upper- and lowermost sites compared to the lag times for snowmelt. A second indication came from the smaller differences in temperature at the time of peak flowering on those sites compared to the summer lapse rate. While these trends are suggestive, they are not very reliable as temperature details are likely to change from year to year. 

By far the best evidence emerged from the thermal sum results. While thermal sums incorporate temperature, they are less affected by seasonal and interannual variation in temperature [12]. When T_BASE_ was held constant at either 1 °C or 5 °C, significant elevational declines in GDD for seven out of twelve flowering phenology metric comparisons were found. In three additional cases, the tendencies were in the same direction but failed to reach significance. These declines allowed peak flowering on the high alpine sites to be situated in the warmest part of the summer. It is instructive to consider when flowering would have taken place on the upper end of *O. squamata’s* range without such phenological adjustment. In the study year, flowering onset would not have occurred until around Dec 16th (fixed T_base_ = 1 °C) and Dec 24th (fixed T_base_ = 5 °C) on Site V. Such dates are fully 21 and 29 days later than the observed flowering onset dates, respectively. Peak flowering would not have occurred until February 11th and 20th which translates to 28 and 37 d later than observed peak flowering. The February dates fell well after the warmest time of the year. Similar elevational declines in thermal sums have recently been reported for high- versus low-elevation populations of alpine species in the northern limestone Alps in the area of Garmisch-Partenkirchen [53]. A parallel situation has been described for species growing on late versus early snow melting sites in Japan [12,63]. Nevertheless, some studies have shown little differentiation of thermal sums over snowmelt gradients [54]; this also seems to be the case in fell field species [15]. The species studied on this occasion, in comparative terms lies somewhere between a snowbed species and a fell field species. To our knowledge, this is the first time an elevational decline in thermal sums has been demonstrated for an alpine species in the South American Andes. 

Unlike in any previous study to our knowledge, GDD was analyzed for an intuitively-developed elevationally-graded series of T_BASE_ temperatures. The logic behind this series was that higher elevation populations of species with long elevational ranges are expected either to adjust plastically or to acquire heritable differences in relation to the elevational dropoff in temperature over the snow-free period in order to maximize growth and reproduction. Various studies have revealed that both plastic and genetic effects are involved in flowering phenology [55,64,65,66]). Under the graded temperature series, no significant elevational trends in thermal sums for any of the flowering metrics were detected. The most obvious interpretation for these results is that flowering could be maintained within the warmest period of the year at all elevations on account of the fact that similar heat sums could be accumulated despite the decreasing temperatures at higher elevations, thanks to the progressive lowering of the temperature threshold for growth. Interestingly, a reduction in the thermal threshold for growth was recently proposed to explain elevational declines in thermal sums found in other alpine species [53]. It has also been mentioned as a possible explanation for a similar trend found in a predominantly lowland annual species with a wide elevational range [55]. In both of these studies, T_BASE_ was held constant. The results for the graded T_BASE_ temperature series can be viewed as a possible mechanistic explanation for the results obtained under fixed T_BASE_ temperatures. An alternative hypothesis to explain the fixed T_BASE_ results sees changes in resource allocation, allowing higher elevation plants to flower after making less vegetative growth. A shorter vegetative period and presumably smaller plants would lead to a lowering of thermal sums at flowering. This explanation does not seem to be applicable to *O. squamata*. Flower number, a good indicator of plant size in this species, showed no tendency to decline with elevation. In fact, on the high alpine, Site IV plants were noticeably larger than on all other sites.

Aside from acting as a cue to the flowering process per se, daylength has been shown to affect the rate of budburst [67]. Temperature-controlled studies in cultivated strawberries have shown that longer days can hasten the time between visible inflorescences and anthesis [68]. Increasing daylength thus would tend to lower the thermal sums associated with flowering phenological stages. In general, the elevational GDD trends for the fixed T_BASE_ temperatures in *O. squamata* were more strongly manifest in the later-expressed phenological metrics. This could indicate that daylength is relevant. The daylength at flowering onset (FF) on the highest site was 0.97 h longer than on the lowest site. By the time peak flowering (FLP) came around, it was actually 0.24 h shorter. However, the speeding up of peak flowering at higher elevations with respect to the physiological expectation could have been imprinted well before the summer solistice was reached. At this point, daylength cannot be rejected as a factor modulating the elevational phenological adjustment observed in *O. squamata.*

Demographic structure could affect GDD if it varies among populations. The frequency of first flowering dates on all sites showed a large early peak and in four cases (excepting the second-highest site) a small late peak. The main peak tended to be less pronounced on the two lower sites. The second peak showed no clear elevational trend. This situation could reflect differences in the amount and timing of first-year recruitment among sites. Germination evidence, necessary to investigate possible demographic effects, was not available.

Despite the flowering phenological adjustment, a two degree centigrade historical temperature gap was seen to characterize the dates of peak flowering on the upper and lower ends of *O. squamata’s* elevational range. This gap is likely to vary in size depending on the variation in snowmelt times among years but is unlikely ever to completely disappear in a mid-season flowering species. Interestingly, such a gap could conceivably close in very early-flowering alpine species if flowering times at higher elevations are pushed well beyond that expected from snowmelt dates. As was predicted, the temperature gap was associated with a decline in relative pollination rates as measured by RPR. Under these circumstances, it was hypothesized that plastically-elongated flower life spans under cooler temperatures could increase the overall probability of pollination, thereby compensating for the lower pollination rates. Good proxy evidence was found for the latter. If so, differences in fruit sets would not be expected over elevation. As expected, good evidence for an effect of temperature around flowers on potential flower longevity was found. However, surprisingly, potential flower longevity did not increase with elevation even after weighting for differences in floral abundance over the flowering season. Moreover, contrary to the expectation under the flower longevity compensation hypothesis, fruit set in the focal species declined with elevation. Reduced resource allocation could be partially responsible for the elevational decline. However, it was notable that the lowest fruit set was on Site IV, which was characterized by the most robust plants. Overall, on two counts, no evidence for the flower longevity compensation hypothesis was forthcoming. Nevertheless, as was shown experimentally (and in natural populations), flower longevity definitely responds plastically to temperature. The only doubtful case was with experimental warming above an already high baseline temperature in the controls on the lowest site. The trend for longer-lived flowers under cooler temperatures in the studied species possibly has more to do with seasonal fluctuations in temperature *within* sites than with the elevational temperature gap *between* sites. Temperatures differences for batches of flowers varied by 9.6 °C to 12.3 °C according to site. It is likely that the life-spans of flowers of *O. squamata* are intrinsically too short to have a measurable impact on increasing the probability of pollination in the upper part of the *O. squamata’s* elevational range. This conclusion leads to the hypothesis that outcrossing species with intrinsically short flower life spans are likely to be less successful in colonizing into very high elevation areas than species with intrinsically longer-lived flowers. However, perhaps such species use other compensatory mechanisms such as increased flower number. Considering the date upon which the maximum number of flowers was open on each individual on a site, average flower number was 21.9 (Site I), 10.7 (Site II), 56.5 (Site III), 89.2 (Site IV), and 18.8 (Site V). However, as the size of plants was not measured, it is difficult to evaluate these data in terms of relative investment in flowers. Plants on Site IV were noticeably larger than on the other sites. 

Two general questions emerge. First, is there any empirical evidence for an elevational decline in the thermal growth threshold in the alpine? Second, if a phenological adjustment occurs either by a lowering of the thermal heat requirement (as seen upon maintaining the lower growth temperature threshold fixed across elevation) or by reducing the thermal threshold for growth but maintaining the same heat requirement (as seen in the graded temperature scenario), how does this come about? With respect to the first question, unfortunately, this is an area of alpine ecology where high-quality information is woefully scarce. The estimated temperature limit for growth at the treeline is 5 °C [62]. At the other extreme of the alpine gradient, growth at 0 °C has been recently reported for a very high alpine species [28]. This comparison suggests a change in the thermal growth threshold is theoretically possible. However, more data is clearly needed. Trees are possibly not comparable to herbaceous species. A number of recent studies have shown that alpine plants develop and flower earlier when transplanted to lower elevation sites or to areas of lower snow accumulation [64,69,70,71]. However, there are cases, where this trend has not been observed [72]. More interestingly, when lower elevation plants are transplanted to higher elevations, they tend to flower later than their high-elevation counterparts; in some cases when high- and mid-elevation plants are simultaneously transplanted to lower elevation, the higher elevation plants flower earlier [64,73]. If T_BASE_ is higher in lower-elevation populations, plants transported to a higher elevation would have to wait more time in the season to flower until temperatures become warmer. Likewise, with a progressively lower T_BASE_ temperature, mid-elevation populations would be less able to take advantage of cooler temperatures at the beginning of the growing season than high elevation populations when both are transplanted to low elevations. If the thermal threshold for growth were the same at all elevations, no difference in flowering times would be expected when plants from different elevations are grown together, unless resource allocation varies elevationally. 

With respect to resource allocation, in a controlled laboratory experiment, high latitude annual *Koenigia islandica* was seen to flower after a similar number of days over an experimental temperature range of 6 to 25 °C. In parallel, thermal sums declined linearly in the 6–18 °C range [74]. The plastic temperature compensation ability shown by this species was explained in terms of a low optimum temperature for growth and productivity and a reduction in plant size at maturity at temperatures below that optimum. In a similar vein, a recent meta-analysis showed that when individuals are transplanted upward they adjusted their traits by decreasing plant growth and number of reproductive units to that of local individuals [75]. These findings incline more towards plants, reducing their thermal sum requirements at flowering by engaging less time in vegetative growth. 

The present study points to some important caveats to bear in mind when searching for trends in flower longevity over elevation. Given the large effort required, flower longevity data are usually collected over a limited number of days in the flowering season. Some data, moreover, are for potential flower longevity, while other data are for actual flower longevity. If floral scenescence is provoked by pollination, these two expressions of flower longevity are not equivalent [32,41]. Moreover, elevational trends in both potential and actual flower longevity could be influenced by flowers living longer under higher soil humidity at higher elevations, independently of cooler temperatures [38,39]. Precaution was taken to filter out these potential complicating factors in the present study. Both potential and actual flower longevity were measured. Flower longevity was determined across the entire flowering season on each site summing to over 70 times and incorporated soil humidity and a proxy for flower size into the analyses. It was also weighted by floral abundance over the flowering season. As no effect of soil humidity or flower size on flower longevity was found in *O. squamata*, it may be concluded that much of the variation in potential flower longevity in the focal species is temperature-related. 

Finally, it may be asked how the focal species will fare under climate change. Reflecting the trend in other high elevation areas, the high central Chilean Andes has warmed by around 0.28 °C per decade as of 1978 [76]. Concomitantly, all of central Chile has seen a consistent decline in precipitation as of the late 1980s [77]. As of 2010, there have 2–5 fewer days of winter snow per year [78] and glacier die back accelerated significantly as [79,80]. Data on flowering phenology in *O. squamata* (under *O. geminata*, syn.) were taken in an adjacent valley almost four decades ago by the lead author and her colleagues [25]. For sites comparable in elevation, flowering occurred earlier in the austral summer of the present study. However, the observation dates in the earlier phenological study were much further apart than in the present study. Thus, this conclusion must be considered tentative. Fruit set measured two decades ago in a subalpine population in the same valley [50] was within the range seen for the subalpine populations and the ecotonal population studied on this occasion. The present study was conducted during a 10 yr drought in central Chile. That little change in fitness seems to have occurred, thus is interesting. 

With increasing temperature, pollination in *O. squamata* could be affected by flowers lasting shorter periods of time as was seen with experimental warming, thereby affecting the success of pollination. Reduced floral longevity was recently documented in a late-season flowering species of Asteraceae in the subalpine belt in the same study area in the severest year of the above-mentioned drought [81]. That year also happened to an exceptionally warm year. Significant reductions in nectar standing crop, flower-head availability, and pollinator visitation were also observed. Reduction in the life-spans of the flower heads would have contributed to the lower number of flower heads. Thus, reductions in flower longevity can affect pollination in two ways: (a) by reducing the opportunity for pollen receipt and, (b) by lowering floral abundance, thereby dampening visual signals. However, in more normal warmer years, shorter-lived flowers in *O. squamata* could be counteracted by flowering occurring earlier in the very long growing season. At the other extreme of its elevational range, *O. squamata* is likely to expand upward, thereby taking advantage of the warmer temperatures for pollination.

## 5. Conclusions

The phenological adjustment documented in this study was fairly subtle. It would not have been picked up from a comparison of the number of days from snowmelt to a flowering phenophase. It was picked up thanks to the wide elevational range occupied by the focal species and the fact that the study sites were fairly homogeneous and not strongly affected by local site variation in snow depth. Needless to say, the calculation of GDD assumes the availability of accurate snow lift dates. The Modis snow cover data used can be imprecise due to cloudy days. Clouds were present for three days on Site I before a full snow lift was detected and one day before it was detected on Site V. However, pushing snowmelt back two more days on Site I than on Site V, if anything, would have strengthened the tendencies found. 

Whether results reflect a plastic modification of the thermal sum requirement/growth temperature threshold versus genetically-based differences cannot be determined. This would require a complex reciprocal transplant experiment involving transplanting all combinations of populations replicated over five sites [55]. This was completely beyond the scope of the present study.

More work is needed on the flowering phenology of late-flowering alpine species. In that, daylength will decline through a greater part of the flowering process in late-seasonal flowering species, the latter could hold the key to understanding the extent to which deep-level physiological effects versus pollination and other biotic factors such a seed maturation mould flowering phenology. In general, few annuals occur in alpine habitats (1). However, they would be well worth investigating so as to eliminate potential demographic effects on GDD. A lack of accurate knowledge on the lower thermal limits for growth in alpine species constitutes a major handicap for advancing in this area of research. Contrary to the prediction of the flower longevity compensation hypothesis, a reduction in fruit set over elevation was found in the study species. Information on the breath of the thermal niches of pollinators is badly needed to obtain a more complete picture of other factors that affect fruit and seed set in alpine species with wide elevational ranges. 

## Figures and Tables

**Table 1:** Site details, snow lift dates, snow-free days, and days from snowmelt to different flowering stages in five populations of *O. squamata.* The flowering stages are: FF = first individual that flowered; FL_50%_ = half of plants in flower; FLP: maximum number of plants in flower; FLSP maximum number of open flowers. N = Number of plants monitored for flowering phenology. Sites I-II are found in the subalpine vegetation belt. Sites IV and V are found in the high alpine belt. Site III lies on the ecotone between the subalpine and high alpine belts.

Site	Lat °S Long °W	Elevation (m)	Mean °C (Range) ^1^	Snow Lift	Snow Free Days	FF	FL_50%_	FLP	FLSP
I (N = 87)	33°21’3” 70°17’49”	2341	12.5 (−0.5–22.6)	31 Aug	250	52	73	106	96
II (N = 44)	33°21’4” 70°15’46”	2596	11.7 (−1.7–21.6)	20 Sept	230	44	60	105	73
III (N = 46)	33°21’3” 70°15’24”	2736	10.4 (−3.0–20.6)	26 Sept	224	38	53	99	111
IV (N = 47)	33°21’2” 70°15’ 02”	2967	8.9 (−4.6–19.2)	30 Sept	220	49	66	98	107
V (N = 61)	33°20’44” 70°14’5”	3151	8.6 (−4.9–18.8)	07 Oct	213	50	62	100	89

^1^ Temperatures are for the total snow-free period (potential growing season) on each site in the study year. Total snow-free days correspond to the period between snow lift and permanent snow the following winter.

**Table 2:** Results of the linear regression of accumulated GDD on elevation for different flowering phenology metrics. Significant values of the regressions are shown in bold. See text for the definition of the six flowering metrics.

Scenario	FF		FF_POP_		FF_50%_		FLP		FLP_POP_		FLSP	
	F_1,4_	P	F_1,4_	P	F_1,4_	P	F_1,4_	P	F_1,4_	P	F_1,4_	P
T_BASE_ = 5 °C	6.654	0.082	19.242	**0.022**	16.705	**0.026**	15.317	**0.030**	10.608	**0.047**	0.42	0.563
T_BASE_ = 1 °C	4.086	0.136	14.072	**0.033**	11.567	**0.042**	14.716	**0.031**	5.088	0.109	1.315	0.335
T_BASE_ graded	0.187	0.695	0.294	0.625	0.206	0.681	0.597	0.496	0.499	0.531	0.398	0.573

**Table 3:** Results of ANCOVA to test for the effect of warming (treatment), flower diameter, and site on flowering longevity in *O. squamata*. Significant P values are shown in bold.

Effect	DFn	DFd	F	P
Flower diameter	1	59	0.002	0.962
Treatment	1	59	9.578	**0.003**
Site	1	59	1.881	0.175
Treatment:Site	1	59	3.569	0.064

**Table 4:** Mixed model ANCOVA for flower longevity. Significant effects are shown in bold. Pollination treatment refers to open-pollinated flowers. Control flowers were pollinator-excluded.

Model: Flower Longevity ~ Treatment + Flower Diameter + Temperature + Soil Moisture + (1 |Site/Batch)					
Fixed Effects	Estimate	Std. Error	Df	t Value	Pr(>|t|)
(Intercept)	4.514	0.318	63.67	14.183	**< 2e-16**
Pollination treatment	−0.656	0.041	781.6	−16.026	**< 2e-16**
Flower diameter	0.012	0.019	691.7	0.645	0.519
Temperature	−0.132	0.014	128.2	−9.762	< 2e-16
Soil moisture	0.0003	0.004	114.8	0.06	0.952

## Data Availability

The data upon which this study is based are available upon request from the Corresponding Author.

## Appendix B

An experiment was carried out in the field to assess the effect of pollination on flower longevity in *O. squamata.* Twenty-five plants were pollinator-excluded on Site V. On each plant, six mature flower buds were marked; three were randomly assigned to a hand-pollination treatment, while the other three served as controls. Treatment flowers were hand cross-pollinated. All plants were pollinator-excluded and the flowers were monitored daily until they closed. The experiment was conducted on long-styled plants, the flowers of which are more amenable to hand-pollination than those of mid- and short-styled plants. Final sample sizes, excluding broken, eaten, and one flower, which did not develop a corolla, were N = 71 (hand-pollination), N = 72 (control). Pollination had a significant effect on the flower life-span (Wilcoxon Rank Sum Test with Continuity Correction: W = 421.5, P = 0.030) (Figure A2).

**Table A1:** Second-order polynomials and significance of regressions used to estimate the number of plants in flower at the beginning and end of the season when field observations were lacking. The number of dates added with flowering at the beginning and end of flowering is given. For the purposes of the equations, days were numbered from 1 = 25 August 2016, onwards. The regressions included the projected number of plants in the flowering phenophase (based on earlier and later observation dates) for one day when heavy snow did not permit making phenological observations across the entire area.

Site	Equation	N	R^2^adj	F	P	Beginning Dates	End Dates
I	y = −0.0178x^2^ + 4.3702x − 189.14	30	0.9654	405.3	2.2e-16	5	0
II	y = −0.0114x^2^ + 2.8758x − 142.71	30	0.9717	499.5	2.2e-16	1	0
III	y = −0.0125x^2^ + 3.1961x − 159.47	31	0.9498	284.5	2.2e-16	1	0
IV	y = −0.0096x^2^ + 2.9519x − 178.49	28	0.8885	108.6	4.702e-13	0	9
V	y = −0.0262x^2^ + 7.1514x − 433.86	19	0.8891	73.14	8.931e-09	2	0
